# An old foe comes swooping back: Whooping cough resurgence—Okay, now what?

**DOI:** 10.1371/journal.ppat.1014157

**Published:** 2026-04-28

**Authors:** Dakota L. Musgrave, C. Buddy Creech

**Affiliations:** Vanderbilt Vaccine Research Program, Department of Pediatrics, Vanderbilt University Medical Center and School of Medicine, Nashville, Tennessee, United States of America; Duke University Medical Center, UNITED STATES OF AMERICA

Whooping cough is a respiratory disease caused by the bacterium *Bordetella pertussis* that can lead to fatal disease in infants and unimmunized individuals. Despite relatively preserved vaccine uptake around the US, there continue to be sporadic outbreaks of pertussis disease that mirror the pre-vaccine cycle of 2–5 years. Data from the US from 2000 to 2016 reveal >300,000 cases of pertussis; approximately 10% of patients were hospitalized. Annual pertussis incidence was highest among infants (~750/100,000), which was >50× higher than other age groups [1]. As pertussis disease incidence has increased overall, so have fatal infections and severe infant disease, which is characterized by respiratory distress, tachycardia, and hyperleukocytosis with mortality as high as 75% [2,3]. During the COVID-19 pandemic, pertussis incidence decreased, but as restrictions lessened, there has been a return of the high disease incidence seen prior to the pandemic that cannot be explained solely by increased detection capabilities or decreased vaccination rates [4].

Pertussis vaccines are widely available around the world. First-generation whole-cell inactivated pertussis vaccines (wP), containing suspensions of killed *B. pertussis* organisms, were widely adopted in the 1940s and greatly reduced infection and transmission. These vaccines were widely recommended and used until the 1990s, when less reactogenic acellular pertussis (aP) vaccines were developed that included key antigens of *B. pertussis* and excluded the lipopolysaccharide endotoxin [5]. As a result, diphtheria, tetanus, and wP vaccines were replaced by diphtheria, tetanus, and aP vaccines in most high-income countries in the 1990s in response to diminished vaccine confidence and excellent antibody responses observed after aP vaccination. While recent estimates indicate a slight decline in aP vaccine coverage, overall pertussis vaccine coverage remains high [6,7].

Current aP formulas contain two to five antigens of *B. pertussis*: pertussis toxin (PT), pertactin (PRN), fimbriae (FIM2/3), and filamentous hemagglutinin (FHA). PT and FHA stimulate antibodies that protect primarily against respiratory challenge; FIM strains stimulate agglutinating antibodies; PRN is an agglutinogen and stimulates antibodies that protect against lethal infant disease [8]. The aP vaccine is currently administered in the United States as a five-dose priming course of DTaP with recommended boosting with Tdap vaccine during adolescence and each pregnancy.

Now, after 30+ years of widespread use of aP vaccines, including the expansion of booster doses among adults and pregnant women, it is increasingly recognized that improvements are needed to provide durable protection against this formidable pathogen. In this concise review, we focus our discussion on pertussis vaccines that may reduce upper respiratory tract infection, transmission, and lower respiratory tract disease. Novel adjuvants, expanded antigen content, and alternative vaccine technologies, such as live attenuated vaccines and less reactogenic whole-cell vaccines, are promising approaches that mimic the robust immunological response elicited by wP vaccines but with reactogenicity profiles similar to current aP vaccines (Fig 1). These novel approaches are crucial to combat the current increases in pertussis incidence.

**Fig 1:**
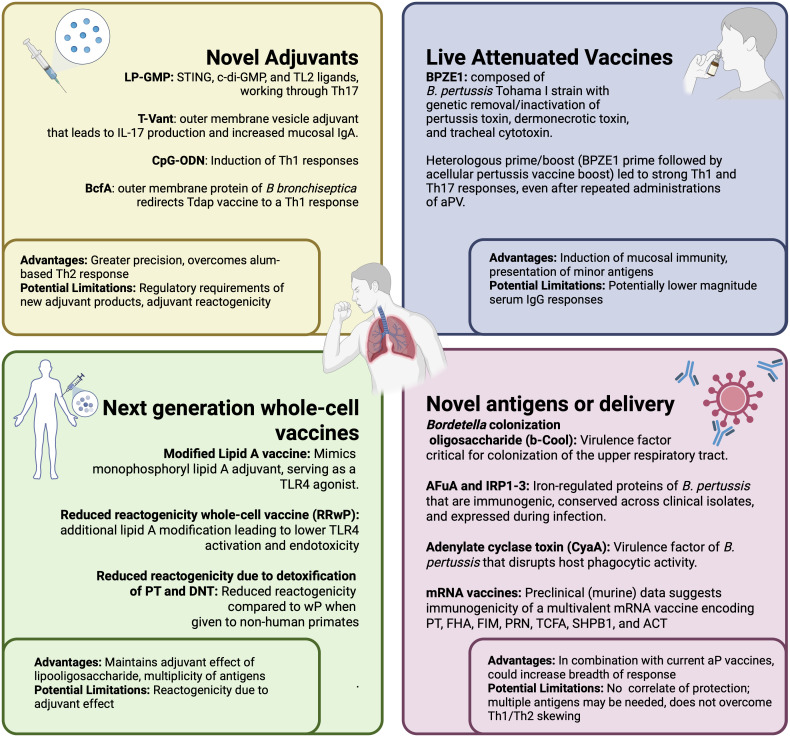
The future of pertussis vaccines. Figure was created in BioRender. Creech, B. (2026) https://BioRender.com/7p9t3fk.

## Immunogenicity of current pertussis vaccines

Three observations are critical to our understanding of pertussis vaccine immunogenicity.

First, while a comprehensive overview of the immune response to aP vaccine is beyond the scope of this review, it is important to note some limitations of current vaccines. Initial responses to DTaP vaccine are blunted in infants born to mothers who received Tdap vaccine during pregnancy [9], though these differences are largely abrogated following booster doses. Immune responses to preschool and adolescent vaccines wane rapidly, with some studies suggesting Tdap effectiveness in adolescents decreasing from 68.8% in the year after vaccination to 8.9% by ≥4 years post-vaccination [10]. Waning immunity has also been observed in pregnant women, with antibody levels rapidly declining by 9–15 months following immunization [1].

Second, the first exposure to pertussis immunization (e.g., aP versus wP) appears to govern the response to pertussis vaccine and control of pertussis once encountered [11,12]. The concept of original antigenic sin, first introduced in 1960 by Dr. Thomas Francis in the context of influenza [13], is now understood as a phenomenon for many pathogens in which a strong anamnestic response to the original exposure constrains the breadth of response to subsequent exposures [14]. While antigenic imprinting to wP or aP antigens may influence subsequent responses, the molecular mechanism for differential immune responses to pertussis vaccines is far from monolithic: linked-epitope suppression, antibody-mediated feedback suppression of naïve B-cells, epigenetic conditioning, rapid antigen clearance due to cross-reactive antibodies, and skewed Th1/Th2 responses may all contribute [12,15,16]. Clinically, these first impressions matter. Sheridan and colleagues observed that children first exposed to wP vaccine had a significantly lower overall disease incidence than those who first received a dose of aP [17]. These results mirror those of Klein and colleagues [18] and Witt and colleagues [19] in the US. As a result of the differential immunogenicity of wP vaccines (and lower cost), many countries have maintained an active immunization program with DTwP despite availability of aP vaccines [20].

Third, the baboon model of pertussis disease, elegantly described by Merkel and colleagues, has given insights into the pathogenesis of *B. pertussis* and the primate response to vaccination [15]. Whole cell pertussis vaccines, similar to natural *B. pertussis* infection, induce Th17 immunity, which is requisite for bacterial clearance at mucosal surfaces [21]. When compared to the wP vaccine, aP vaccines preferentially lead to Th2 responses, which are less efficacious and lead to a shorter duration of protection [15]. As a result, aP vaccines protect against disease, but do not prevent infection of the upper respiratory tract; aP-vaccinated animals remained consistently colonized after infection for 35 days, compared to 18 days in wP-vaccinated animals [15]. Thus, aP vaccines might contribute to more persistent transmission, including among asymptomatic individuals [22,23].

## Novel adjuvants may overcome some of the limitations of aP vaccines

Several novel adjuvants have been considered for safety and efficacy in combination with the acellular pertussis vaccine [24]. Broadly, the goal of these adjuvants is to redirect the weaker Th2 immune response that is characteristic of current aP vaccines to the more robust Th1/Th17 response of the wP vaccine. Results from studies of several candidates show that these novel adjuvants redirected immunogenicity without the same reactogenicity concerns.

One such candidate is the novel adjuvant combination LP-GMP, which, when introduced intranasally with a novel aP formulation, confers long-term protection against both nasal and respiratory infection through IL-17-secreting CD4+ tissue resident T cells in mice [25]. This combination of bacterial DNA sensor STING, c-di-GMP, and TLR2 ligands could both reduce nasal infection and overcome waning immunity through a Th17 response [25].

Outer membrane vesicles (OMVs) are secreted by Gram-negative bacteria and possess inherent adjuvanticity through TLR2 and TLR4 activation [26]. OMVs derived from *B. pseudomallei* have been identified as a safe and effective vaccine adjuvant due to naturally attenuated cytotoxicity and have been employed in several vaccines for respiratory pathogens [27]. T-Vant is a novel OMV-based adjuvant derived from *B. pseudomallei* for use in combination with an intranasal aP priming dose for pertussis; intranasal immunization with T-Vant-adjuvanted-aP vaccines demonstrates sterilizing immunity in the nasopharynx associated with IFN-γ and Il-17 producing CD4+ T cells [27]. In addition to rapid NP clearance, aP-T-Vant immunized mice had significantly higher mucosal IgA titers than naïve controls as well as higher serum IgG than DTaP-immunized mice, making this another promising candidate to redirect the immunological response of current aP vaccines. Other vaccine candidates use *B. pertussis*-derived OMVs, which require an additional genetic modification to attenuate OMV-associated lipid A cytotoxicity, inducing a strong Th1/Th17-related local immune response when delivered intranasally [28]. OMVs currently include a diverse range of adjuvant candidates for novel pertussis vaccines.

Additional adjuvants have been developed as candidates for adult boosting, which could serve as solutions to waning immunity in aP-primed individuals. The TLR9 agonist CpG oligodeoxynucleotide (CpG-ODN) is an adjuvant that stimulates a strong Th1 immune response, is being evaluated as a component for many vaccines, and is included in the hepatitis B vaccine, Heplisav-B [29]. In pertussis, specifically, CpG-ODN enhances specific humoral and cellular responses through Th1 induction regardless of PRN deficiency, which is important as PRN(−) strains become more prevalent in aP vaccinated areas [30,31]. Tdap boosters adjuvanted with CpG 1018 conferred significantly higher GMCs of anti-PT antibodies and anti-PT neutralizing antibodies when compared to the currently licensed Boostrix vaccine [30].

Finally, inclusion of BcfA, an outer membrane protein from *Bordetella bronchiseptica,* with commercial alum-adjuvanted Tdap (Boostrix) provides better protection against infection through redirection from Th2 to a primarily Th1 response in mice [32]. These booster adjuvants could offer the reprogramming necessary to account for the shorter durability of aP priming.

## Additional vaccine antigen targets

Notably, none of the antigens included in current aP vaccines confer direct protection against upper respiratory tract colonization, which, as shown in non-human primate models, directly contributes to continued infection and transmission of pertussis [15]. Additional vaccine antigen targets could improve immunogenicity of novel vaccines.

Early findings have identified the glycan product of the *Bordetellae* colonization oligosaccharide (b-Cool) locus as playing a critical role in nasal colonization in multiple *Bordetella* species, including *B. pertussis.* Disruption of b-Cool pathways was found to hinder nasal colonization and transmission in mice, suggesting that inhibition of b-Cool biosynthesis offers a potential target for reduced transmission [33].

AFuA (Bp1605) and IRP1–3 are iron-regulated proteins of *B. pertussis* identified by Alvarez Hayes and colleagues [34]. Both proteins are surface-exposed, conserved across clinical isolates, and expressed during infection. Murine studies confirm opsonophagocytic antibody responses and protection against respiratory challenge.

Adenylate cyclase toxin (CyaA) is a virulence factor of *B. pertussis* that undermines innate immunity by dysregulating cAMP signaling in host phagocytes. Murine models suggest that a recombinant form of ACT that lacks enzymatic activity enhanced protection against intranasal challenge of *B. pertussis* when administered with aP vaccine and promoted Th1-biased and CD8+ T-cell responses [35].

Additionally, mRNA vaccines offer an adaptable and affordable option for inclusion of new antigens. Recent murine studies have shown that a multivalent mRNA vaccine encoding for the aP vaccine components PT, FHA, FIM, and PRN as well as known toxins TCFA, SHPB1, and CyaA is immunogenic and provides protection upon challenge with pertussis [36].

Loss of vaccine antigens under selective pressure from the aP vaccine is hypothesized to contribute to the resurgence of transmission and infection [37]. A 2024 pertussis outbreak in China was recently linked to the prevailing macrolide-resistant type ptxsP3 strain; lack of treatment options likely contributed to this most recent outbreak [38,39]. Pertactin and filamentous hemagglutinin-deficient isolates exist; however, no evidence suggests that these strains are linked to aP vaccine failure [31,40]. Nonetheless, the identification and inclusion of additional highly conserved antigens for next-generation acellular vaccines should remain a priority in combatting future circulation as pertussis continues to evolve [41,42].

## Live attenuated vaccine candidates

The intranasal live attenuated vaccine BPZE1 was developed from *B. pertussis* Tohama I strain that was modified using either genetic removal or inactivation of 3 major *B. pertussis* toxins: pertussis toxin, dermonecrotic toxin, and tracheal cytotoxin [43,44]. Preclinical and early phase clinical data demonstrate that the vaccine is safe and induces both mucosal and systemic immune responses that vary by dose administered. Mielcarek and colleagues showed that adult and infant mice vaccinated with BPZE1 are protected against challenge with *B. pertussis* [44]. Moreover, Feunou and colleagues showed that a heterologous prime/boost strategy (BPZE1 prime followed by acellular pertussis vaccine boost) led to strong Th1 and Th17 responses, even after repeated administrations of aP vaccine [45]. Non-human primate data showed that nasopharyngeal colonization could be induced in all animals, which resolved by 45 days post-inoculation [46]. Systemic illness was not observed, nor were changes in white blood cell counts (a hallmark of pertussis disease), and the vaccine led to systemic IgG and mucosal IgA responses to PT, FHA, and PRN. Most importantly, BPZE1 induced protection against virulent *B. pertussis* (D420 strain) challenge.

The clinical development of BPZE1 has now entered Phase 3 clinical trial readiness, based principally on successful safety and immunogenicity studies in healthy adults, as well as compelling data from a controlled human infection model (CHIM) of pertussis [47,48]. In the CHIM, intranasal BPZE1 substantially reduced infection after challenge with *B. pertussis.* Moreover, solicited and unsolicited adverse events were similar between study groups.

## Reduced reactogenicity whole-cell vaccines

*Bordetella* endotoxins are highly variable across strains and explain the reactogenicity of original wP vaccines. While the precise mechanism of *B. pertussis* endotoxin-mediated reactogenicity in humans is not fully elucidated, mouse models suggest that induction of TLR4 inflammatory pathways contributes to protection [5]. The characterization of the lipid A moiety of lipopolysaccharide (LPS) provides important context for this mechanism. Lipid A anchors LPS to the Gram-negative outer leaflet of pertussis and binds to Toll-like receptor 4 (TLR4) to stimulate the innate immune response [49]. Two distinct pathways, MyD88-dependent and TRIF-dependent, can be activated to produce cytokines which either cause LPS to act as an endotoxin or adjuvant [50,51].

The manipulation of lipid A structure could contribute to novel aP-adjuvanted vaccines as well as less reactogenic wP vaccines through modulation of TLR4 pathways [50]. Using natural biosynthetic pathways, lipid A modifications have been determined which, in combination, achieve lower reactogenicity for wP vaccines. One lipid A variation mimics monophosphoryl lipid A (MPLA) adjuvant structure by modifying acyl chains as well as phosphorylation state of lipid A [50]. A reduced reactogenicity whole-cell pertussis vaccine (RRwP) including such lipid A modifications with lower TLR4 activation and endotoxicity, as well as detoxification of pertussis toxin and dermonecrotic toxin (DNT), has shown reduced reactogenicity as compared to original wP vaccines when introduced intramuscularly in baboons [52]. RRwP vaccines are promising low-cost, immunologically robust vaccine candidates.

Recent preclinical evidence of an antibiotic-inactivated *Bordetella pertussis* (AIBP) vaccine platform suggest that these vaccines may induce immunity necessary to prevent local colonization without the systemic inflammation that makes the original wP vaccines challenging [53]. When administered via aerosol or intranasally, the AIBP platform induced Il-17 producing CD4+-T_RM_ cells necessary for nasal clearance and also demonstrates a marked increase in IgA in respiratory tissue [25]. While original wP vaccines lead to increased pro-inflammatory cytokines, CRP, and neutrophil concentrations in serum, AIBP vaccine showed no such systemic reactivity. Notably, this platform also seems to account for the issue of linked epitope suppression; aP-primed mice showed no differences in the AIBP-induced Th1/Th17 response. Ongoing development of AIBP will be required to ensure that safety and immunogenicity are maintained, either in non-human primate studies or CHIM.

Despite current efforts to vanquish this old foe, recent epidemiologic trends make one thing clear: something needs to change if we want to say goodbye to pertussis. Disease outbreaks continue to occur despite high vaccine uptake in the US and across the globe. Current wP and aP vaccines have laid a firm foundation for the control of whooping cough, and we recognize that new approaches are needed to address *B. pertussis* infection and transmission.
